# Inflammatory Protein Panel: Exploring Diagnostic Insights for Peripheral Artery Disease Diagnosis in a Cross-Sectional Study

**DOI:** 10.3390/diagnostics14171847

**Published:** 2024-08-24

**Authors:** Ben Li, Rakan Nassereldine, Farah Shaikh, Houssam Younes, Batool AbuHalimeh, Abdelrahman Zamzam, Rawand Abdin, Mohammad Qadura

**Affiliations:** 1 Division of Vascular Surgery, St. Michael’s Hospital, Unity Health Toronto, University of Toronto, Toronto, ON M5B 1W8, Canada; benx.li@mail.utoronto.ca (B.L.); farah.shaikh@unityhealth.to (F.S.); abdelrahman.zamzam@gmail.com (A.Z.); 2 Department of Surgery, University of Toronto, Toronto, ON M5S 1A1, Canada; 3 Temerty Centre for Artificial Intelligence Research and Education in Medicine (T-CAIREM), University of Toronto, Toronto, ON M5S 1A1, Canada; 4 Institute of Medical Science, University of Toronto, Toronto, ON M5S 1A1, Canada; 5 Division of Vascular Surgery, American University of Beirut Medical Center, Beirut 1107 2020, Lebanon; rakan.nassereldine.md@gmail.com; 6 Heart, Vascular, & Thoracic Institute, Cleveland Clinic Abu Dhabi, Abu Dhabi 112412, United Arab Emirates; hkyounesmd@gmail.com (H.Y.); abuhalb@ccf.org (B.A.); 7 Department of Medicine, McMaster University, Hamilton, ON L8S 4L8, Canada; rawand.abdin@medportal.ca; 8 Li Ka Shing Knowledge Institute, St. Michael’s Hospital, Unity Health Toronto, University of Toronto, Toronto, ON M5B 1W8, Canada

**Keywords:** peripheral artery disease, biomarkers, diagnosis

## Abstract

Cytokine-induced neutrophil chemoattractant 1 (CINC-1), a cluster of differentiation 95 (CD95), fractalkine, and T-cell immunoglobulin and mucin domain 1 (TIM-1) are circulating proteins known to be involved in inflammation. While their roles have been studied in neurological conditions and cardiovascular diseases, their potential as peripheral artery disease (PAD) biomarkers remain unexplored. We conducted a cross-sectional diagnostic study using data from 476 recruited patients (164 without PAD and 312 with PAD). Plasma levels of CINC-1, CD95, fractalkine, and TIM-1 were measured at baseline. A PAD diagnosis was established at recruitment based on clinical exams and investigations, defined as an ankle-brachial index < 0.9 or toe-brachial index < 0.67 with absent/diminished pedal pulses. Using 10-fold cross-validation, we trained a random forest algorithm, incorporating clinical characteristics and biomarkers that showed differential expression in PAD versus non-PAD patients to predict a PAD diagnosis. Among the proteins tested, CINC-1, CD95, and fractalkine were elevated in PAD vs. non-PAD patients, forming a 3-biomarker panel. Our predictive model achieved an AUROC of 0.85 for a PAD diagnosis using clinical features and this 3-biomarker panel. By combining the clinical characteristics with these biomarkers, we developed an accurate predictive model for a PAD diagnosis. This algorithm can assist in PAD screening, risk stratification, and guiding clinical decisions regarding further vascular assessment, referrals, and medical/surgical management to potentially improve patient outcomes.

## 1. Introduction

Affecting more than 200 million patients globally, peripheral artery disease (PAD) involves chronic atherosclerosis in the lower extremity arteries [1,2]. Despite being a leading cause of amputation and death, PAD is underdiagnosed and undertreated worldwide [3]. A key challenge is the absence of standardized diagnostic biomarkers in clinical practice that can effectively identify high-risk individuals who could benefit from additional vascular work-up and treatment.

Cytokine-induced neutrophil chemoattractant 1 (CINC-1) [4], a cluster of differentiation 95 (CD95) [5], fractalkine [6], and T-cell immunoglobulin and mucin domain 1 (TIM-1) [7] are circulating proteins that have been extensively studied in relation to cardiovascular diseases such as coronary artery disease (CAD), cerebrovascular disease (CVD), and PAD. This study focused on these four proteins due to their well-documented associations with various cardiovascular conditions, indicating their potential relevance to PAD [4,5,6,7]. While previous research has established links between these proteins and cardiovascular disorders, few studies have specifically investigated their diagnostic utility for PAD [4,5,6,7]. Pathophysiological studies have shown that PAD shares common mechanisms with CAD, including thrombosis, inflammation, dyslipidemia, and microvascular disease, highlighting a significant correlation between the two conditions [8]. Moreover, existing research has predominantly examined individual proteins without exploring a combined panel of these circulating proteins for diagnostic purposes.

PAD is a chronic and multifaceted disorder influenced by various metabolic pathways [9]. Therefore, integrating a panel of biomarkers may improve diagnostic precision compared to assessing individual proteins alone [9]. Our hypothesis is that by integrating biomarker information with clinical features linked to PAD progression, it is feasible to create highly accurate predictive algorithms for diagnosing PAD [10,11,12]. This research aimed to identify PAD-specific biomarkers and merge clinical and biomarker data to develop a diagnostic tool for PAD.

## 2. Materials and Methods

### 2.1. Ethical Approval

Approval for this project was obtained from the Unity Health Toronto Research Ethics Board on 8 February 2017 (REB # 16-365). Prior to participation, all participants provided written informed consent, and all procedures adhered to the Declaration of Helsinki [13].

### 2.2. Design

This cross-sectional diagnostic study was conducted and reported following the guidelines outlined in the Transparent Reporting of a Multivariable Prediction Model for Individual Prognosis or Diagnosis + Artificial Intelligence (TRIPOD + AI) statement [14].

### 2.3. Patient Recruitment

This study involved the cross-sectional recruitment of patients, including those without and with PAD, who attended ambulatory clinics at Unity Health Toronto between September 2020 and February 2022. Diagnosis of PAD was based on a toe-brachial index (TBI) < 0.67 or ankle-brachial index (ABI) < 0.9, along with pedal pulses that were absent or diminished [15]. Non-PAD was defined as a TBI of 0.67 or higher, an ABI of 0.9 or higher, and normal pedal pulses [15]. The exclusion criteria included patients with elevated troponin levels, acute coronary syndrome, or acute limb ischemia in the preceding 90 days.

### 2.4. Baseline Characteristics

The baseline patient features recorded were gender, age, and cardiovascular risk factors: hypertension (defined as a diastolic blood pressure ≥ 80 mmHg, systolic blood pressure ≥ 130 mmHg, or use of antihypertensives [16,17]), dyslipidemia (defined as triglycerides > 1.7 mmol/L, total cholesterol > 5.2 mmol/L, or use of lipid-lowering medications [16,17]), diabetes (defined as use of antidiabetic medications or hemoglobin A1c ≥ 6.5% [16,17]), smoking habits, presence of coronary artery disease (CAD), congestive heart failure (CHF), previous stroke, and use of cardiovascular medications including statins, acetylsalicylic acid (ASA), angiotensin II receptor blockers (ARB) or angiotensin-converting enzyme inhibitors (ACE-I), calcium channel blockers, beta-blockers, furosemide or hydrochlorothiazide, insulin, or oral antihyperglycemic agents.

### 2.5. Measurement of Plasma Protein Levels

Blood samples were acquired from the participants via the median cubital vein by a trained phlebotomist and were collected in citrate tubes. The plasma was isolated through centrifugation, aliquoted, and stored at −80 °C until analysis without undergoing freeze–thaw cycles. Upon analysis, the plasma samples were thawed at room temperature, and concentrations of four circulating proteins (CINC-1, CD95, fractalkine, and TIM-1) were measured in duplicate using the Luminex assay kit (Bio-Techne, Minneapolis, MN, USA) [18]. These proteins were selected for assessment because of their involvement in metabolic processes related to atherosclerosis and the development/progression of cardiovascular diseases. Prior to sample analysis, Fluidics Verification and Calibration bead kits (Luminex Corp; Austin, TX, USA) [19] were utilized for the calibration of the MagPix analyzer (Luminex Corp; Austin, TX, USA) [20]. To minimize variability between the assays, all analyses were performed on a single day. The inter- and intra-assay coefficients of variability were below 10%. Data acquisition and analysis for each protein involved capturing at least 50 beads per sample using Luminex xPonent software version 4.3 [21].

### 2.6. Development and Evaluation of the Diagnostic Model

The chosen prediction algorithm was random forest, an ensemble learning method that works through several decision trees [22]. We used decision trees to segment cohorts into branches and used multiple variables to build predictive algorithms for the outcome of interest [23]. Since random forest is non-parametric, it is adept at handling complex datasets efficiently [23]. Random forest is commonly used in the literature and performs well with healthcare data [24,25,26]. The application of the random forest model in our study specifically relates to its ability to differentiate individuals with vs. without PAD through rigorous diagnostic training.

The data were split randomly into training (70%) and test (30%) sets. Ten-fold cross-validation was used to train the random forest model to predict the PAD diagnosis. Model input features were clinical features, including sex, age, dyslipidemia, hypertension, diabetes, smoking history, CHF, CAD, history of stroke, statins, ASA, ACE-I or ARB, calcium channel blocker, beta-blocker, furosemide or hydrochlorothiazide, insulin, and oral antihyperglycemic agents, as well as plasma CINC-1, CD95, and fractalkine levels. After training, unseen test set data were used to assess the model’s performance using the primary evaluation metric of the area under the receiver operating characteristic curve (AUROC) [27]. The importance of predictive features in the model was assessed by calculation of the variable importance score (gain), which measures the influence of individual variables on prediction accuracy [28].

### 2.7. Statistical Analysis

The baseline features were reported as means (standard deviation [SD]) or numbers (%). Differences between groups were evaluated using an independent *t*-test (continuous variables) or a chi-square test (categorical variables). Protein levels were compared between the PAD and non-PAD patients using an independent *t*-test (normal distribution) or Mann–Whitney U test (non-normal distribution). Proteins showing differential expression between groups were selected for inclusion in the predictive model. Multivariable regression analysis was conducted, adjusting for all baseline features to evaluate the independent association between the protein levels and PAD diagnosis. The predictive performance of the model was evaluated for distinguishing PAD from non-PAD patients using the AUROC as the primary metric. Statistical significance was set at a two-tailed *p*-value of < 0.05. All analyses were performed using SPSS software version 23 (SPSS Inc., Chicago, IL, USA) [29].

## 3. Results

### 3.1. Patients

This study included 476 patients (164 without PAD and 312 with PAD). The PAD patients were older (mean age 71 [SD 10] vs. 65 [SD 12] years, *p* < 0.001) and exhibited a higher prevalence of hypertension (82% vs. 59%, *p* < 0.001), dyslipidemia (84% vs. 61%, *p* < 0.001), diabetes (42% vs. 21%, *p* < 0.001), CAD (38% vs. 21%, *p* < 0.001), previous stroke (16% vs. 8%, *p* = 0.011), and past/current smoking (80% vs. 64%, *p* = 0.002). Additionally, patients with PAD were more frequently prescribed medications for cardiovascular risk reduction, including ASA (80% vs. 60%, *p* < 0.001), statins (73% vs. 57%, *p* < 0.001), beta-blockers (41% vs. 30%, *p* = 0.001), and ACE-I/ARB (66% vs. 45%, *p* = 0.001) (Table 1).

### 3.2. Plasma Protein Concentrations

Among the four proteins examined, three showed statistically significant elevations in PAD vs. non-PAD patients as follows: CINC-1 (79.42 [SD 69.51] vs. 63.08 [SD 41.99] pg/mL, *p* < 0.001), CD95 (5.48 [SD 3.12] vs. 4.56 [SD 1.94] pg/mL, *p* = 0.001), and fractalkine (1093.42 [SD 1063.36] vs. 904.21 [SD 423.76] pg/mL, *p* = 0.02) (Table 2). Therefore, these three proteins (CINC-1, CD95, and fractalkine) were selected for further analysis.

### 3.3. Associations between Proteins and PAD Diagnosis

There were significant associations between the following proteins and PAD diagnosis after adjusting for all of the baseline characteristics (sex, age, dyslipidemia, hypertension, past/current smoking, diabetes, CAD, CHF, previous stroke, statins, ASA, beta-blocker, ACE-I or ARB, calcium channel blocker, furosemide or hydrochlorothiazide, insulin, and oral antihyperglycemic agents): CD95 (adjusted OR 2.63 [95% CI 1.73–3.99], *p* = 0.001) and fractalkine (adjusted OR 2.58 [95% CI 1.63–3.90], *p* = 0.001) (Table 3).

### 3.4. Model Performance

**Figure 1:**
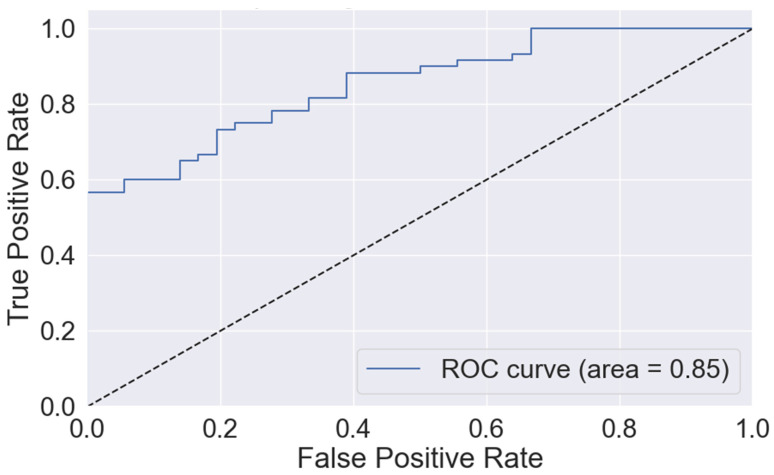
Receiver operating characteristic curve for random forest model, including clinical features and 3-protein biomarker panel [cytokine-induced neutrophil chemoattractant 1 (CINC-1), cluster of differentiation 95 (CD95), and fractalkine] in predicting 2-year diagnosis of peripheral artery disease in the testing cohort. Area represents the area under the receiver operating characteristic curve (AUROC).

**Figure 2:**
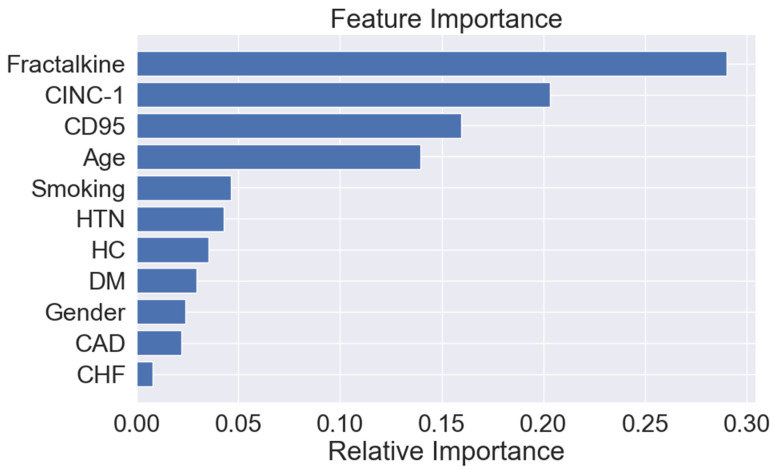
Variable importance scores (gain) for the clinical characteristics and 3-protein biomarker panel [cytokine-induced neutrophil chemoattractant 1 (CINC-1), cluster of differentiation 95 (CD95), and fractalkine] used as input features for random forest model for peripheral artery disease diagnosis. Note: a higher score signifies a greater importance in influencing an overall prediction. Abbreviations: cytokine-induced neutrophil chemoattractant 1 (CINC-1), cluster of differentiation 95 (CD95), congestive heart failure (CHF), coronary artery disease (CAD), diabetes mellitus (DM), hypercholesterolemia (HC), and hypertension (HTN).

The model achieved excellent performance in predicting a PAD diagnosis (AUROC 0.85, Figure 1). The most important predictive features for the model were (1) fractalkine, (2) CINC-1, (3) CD95, (4) age, and (5) current/past smoking (Figure 2).

## 4. Discussion

### 4.1. Key Findings

Herein, we identified a 3-protein PAD biomarker panel consisting of CINC-1, CD95, and fractalkine. Utilizing the clinical features and plasma biomarker levels, we developed a robust model that accurately predicts a PAD diagnosis. Among the four circulating proteins analyzed, CINC-1, CD95, and fractalkine were found to be elevated in PAD vs. non-PAD patients. Our predictive model, incorporating these biomarkers along with the clinical features, demonstrated a strong performance for a PAD diagnosis. Feature importance analysis highlighted the biomarker levels as the most influential predictors, underscoring the relevance of these biomarkers in predicting PAD outcomes. The significance of CINC-1, CD95, and fractalkine lies in their potential roles in PAD pathogenesis, attributed to their pro-inflammatory, atherogenic, and microvascular effects [30,31,32]. Given their importance in PAD diagnosis, further research is needed to understand the biological mechanisms linking these biomarkers to PAD progression. This endeavor aims to advance targeted diagnostics and therapeutic strategies for PAD management. An accurate prediction of a PAD diagnosis is suggested based on the findings from this cross-sectional study, and future prospective studies are needed to confirm our results.

### 4.2. Comparison to Existing Literature

Ross and colleagues built a model aimed at predicting major adverse cardiac and cerebrovascular events (MACCE) in patients with PAD using electronic health records data, achieving an AUROC of 0.81 [33]. However, a limitation was the absence of biomarker data as input features, which we addressed by integrating biomarker information into our models. Focusing on a PAD diagnosis, our approach yielded excellent performance, with an AUROC of 0.85. This highlights the advantage of incorporating biomarker information in predictive modeling, which may enhance the accuracy compared to relying solely on clinical characteristics.

### 4.3. Explanation of Findings

Our study identified three biomarkers—CINC-1, CD95, and fractalkine—that were significantly elevated in patients with PAD, underscoring their importance as predictors of a PAD diagnosis. These proteins play pivotal roles in several cardiovascular pathways. CINC-1, known for its role in mediating inflammatory processes in animal models, contributes significantly to atherosclerosis, which is important for PAD development [4]. Inhibition of CINC-1 has shown promise in reducing cardiac inflammation and damage in conditions like pulmonary embolism [30]. CD95 is a death receptor involved in apoptosis induction on engagement by the Fas ligand [31]. CD8 T-cells expressing CD95 demonstrate pro-atherogenic effects, contributing to cardiovascular disease [5]. Fractalkine is a large chemokine of 373 amino acids, which is implicated in atherosclerosis development and has been demonstrated to be elevated in patients with cardiovascular diseases [6]. Specifically, fractalkine has been demonstrated to be associated with microvascular obstruction in patients undergoing percutaneous coronary intervention [32]. Furthermore, inhibition of fractalkine in murine models may reduce the infarct size, inflammation, and intramyocardial hemorrhage [32]. Altogether, these discoveries describe potential mechanisms through which these three biomarkers may be involved in PAD development. Importantly, our understanding of the pathophysiology of PAD has been recently enriched by Miceli and colleagues (2022) regarding the important role of inflammation and its cross-talk with coagulation [34]. Specifically, Miceli et al. (2022) reviewed the molecular contribution of inflammation and the coagulation system on PAD pathogenesis, including the molecular similarities and differences between atherogenesis in PAD and CAD [34]. The inflammatory markers analyzed in this study may play an important role in these cross-talk pathways to contribute to PAD pathogenesis. Our diagnostic model demonstrated strong performance for multiple reasons. In contrast to traditional statistical techniques, such as logistic regression, which assumes that there is a linear relationship between the covariates and outcomes, advanced machine learning techniques can better capture complex, nonlinear interactions within healthcare data [35,36]. This flexibility is crucial given the multifactorial nature of patient outcomes in healthcare [37]. Machine learning’s ability to automate tasks, understand relationships that are nonlinear, and provide highly accurate predictions makes it particularly advantageous in models that include biomarker data, as proteins may interact in intricate ways to influence disease processes [38]. The random forest model employed in our study performed well, likely because of its ensemble learning method, which aggregates multiple decision trees [39]. This method decreases variance and handles large datasets effectively while minimizing overfitting [39]. Overall, our work highlights the benefits of integrating biomarkers into predictive models, surpassing the predictive power of relying solely on clinical data. This approach not only saves time but also holds promise for use by primary care physicians in routine practice. Given that PAD involves multiple biological pathways and shares risk factors with other cardiovascular diseases like CAD and CVD, our study underscores the importance of a comprehensive diagnostic approach [40]. Additionally, our work highlights the theory of polyvascular disease due to clinically evident atherosclerosis in multiple arterial beds and the fact that PAD may increase the risk of adverse outcomes in other disease conditions such as CAD and CVD [8]. Therefore, these cardiovascular conditions may benefit similarly from the types of diagnostic models described in this study [8]. Our work emphasizes the potential for advanced predictive models to enhance risk prediction tools for PAD by leveraging both clinical and biomarker data effectively.

### 4.4. Implications

Our predictive models have significant potential to guide clinical decisions in several contexts. First, the model may help screen individuals for asymptomatic PAD [41]. Individuals who screen positive for PAD may receive additional vascular evaluation, such as an arterial duplex ultrasound, to assess their blood flow and confirm a PAD diagnosis [42]. Those identified to be at low risk of PAD may receive ongoing care from their family physician, focusing on risk factor optimization through treatment options, including statins, ASA, and lifestyle adjustments [43]. Conversely, patients deemed high risk for PAD should promptly receive referrals to vascular surgeons for additional evaluation and management [44]. Vascular surgeons can use the algorithm alongside clinical judgment to identify high-risk patients who may benefit from additional imaging to delineate vascular anatomy and the severity of disease [45], as well as low-dose rivaroxaban [46] or limb salvage interventions [47,48]. Overall, the tool may enhance PAD care in both specialist and generalist environments. It can streamline screening for PAD and risk stratification, potentially reducing unneeded referrals, improving outcomes, and lowering costs [49].

### 4.5. Limitations

While these findings have important implications for PAD screening and early management, we acknowledge several limitations in our study. Firstly, it was performed at one center, and further validation in other settings is needed to confirm its generalizability. Secondly, given that machine learning methods require large amounts of data for accurate performance, our sample size of 476 patients may be limited, and future studies with larger sample sizes may allow for the development of more robust models. Thirdly, patients with PAD in this study had a higher prevalence of CAD than those without PAD. Given that the plasma concentrations of the inflammatory markers analyzed in this study may be influenced by the presence of CAD, this may be a confounding variable. We controlled for the presence of CAD and other risk factors using multivariable regression analysis and demonstrated independent associations between the inflammatory proteins and PAD, highlighting the specificity of these markers for a PAD diagnosis. However, future randomized studies may further reduce the risk of selection bias and allow for a more robust evaluation of our models. Fourthly, the identified biomarkers are currently used primarily in the research setting. Additional work is required to establish the clinical value and feasibility of incorporating these biomarkers into routine PAD care.

## 5. Conclusions

Herein, a 3-biomarker panel (CINC-1, CD95, and fractalkine) was identified for PAD and incorporated with clinical characteristics to build a model that effectively predicts a PAD diagnosis. Our model demonstrates the potential for supporting PAD screening and risk stratification, facilitating an earlier diagnosis and personalized treatment of the condition. Prompt modification of medical risk factors could improve patient outcomes. Specifically, the model may identify high-risk individuals who should be referred for additional vascular work-up and potentially receive more aggressive medical/surgical interventions. The adaptive learning and automated features of our algorithms could further enhance their application in clinical practice, potentially leading to advancements in PAD patient care. Additionally, our research underscores the importance of additional work to elucidate the biological roles of CINC-1, CD95, and fractalkine in PAD pathogenesis, offering insights for developing personalized diagnostic and therapeutic strategies.

## Figures and Tables

**Table 1:** Baseline characteristics.

	PAD (*n* = 312)	Non-PAD (*n* = 164)	*p*
Age, mean (SD)	71 (10)	65 (12)	<0.001
Female sex	109 (35)	67 (41)	0.204
Dyslipidemia	263 (84)	100 (61)	<0.001
Hypertension	257 (82)	96 (59)	<0.001
Diabetes	131 (42)	34 (21)	<0.001
Current smoking	78 (25)	35 (21)	0.002
Past smoking	171 (55)	71 (43)	0.001
Congestive heart failure	11 (4)	4 (2)	0.519
Coronary artery disease	118 (38)	34 (21)	<0.001
Previous stroke	51 (16)	13 (8)	0.011
Statin	229 (73)	93 (57)	<0.001
Acetylsalicylic acid	251 (80)	99 (60)	<0.001
Beta-blocker	134 (41)	50 (30)	0.001
ACE-I/ARB	216 (66)	74 (45)	0.001
Hydrochlorothiazide or furosemide	41 (13)	17 (10)	0.190
Calcium channel blocker	82 (25)	34 (21)	0.079
Insulin	22 (7)	6 (4)	0.255
Oral antihyperglycemic agent	24 (8)	8 (5)	0.201

Values reported as *n* (%) unless stated otherwise. Abbreviations: ARB (angiotensin II receptor blocker), ACE-I (angiotensin-converting enzyme inhibitor), SD (standard deviation), and PAD (peripheral artery disease).

**Table 2:** Plasma protein concentrations.

	Non-PAD (*n* = 164)		PAD (*n* = 312)		
	Mean	Standard Deviation	Mean	Standard Deviation	*p*
CINC-1	63.08	41.99	79.42	69.51	<0.001
CD95	4.56	1.94	5.48	3.12	0.001
Fractalkine	904.21	423.76	1093.42	1063.36	0.020
TIM-1	22.48	8.58	27.04	19.51	0.125

Protein concentrations reported in pg/mL. Abbreviations: cytokine-induced neutrophil chemoattractant 1 (CINC-1), cluster of differentiation 95 (CD95), and T-cell immunoglobulin and mucin domain 1 (TIM-1).

**Table 3:** Associations between proteins and peripheral artery disease diagnosis.

	Unadjusted Odds Ratio [95% CI]	*p*-Value	Adjusted Odds Ratio [95% CI] *	*p*-Value
CINC-1	1.36 [0.05–3.33]	0.503	3.10 [0.33–6.39]	0.322
CD95	2.47 [2.03–3.25]	0.001	2.63 [1.73–3.99]	0.001
Fractalkine	2.73 [1.99–4.14]	0.001	2.58 [1.63–3.90]	0.001

* Adjusted for age, sex, hypertension, diabetes, dyslipidemia, current/past smoking, congestive heart failure, coronary artery disease, previous stroke, acetylsalicylic acid, statin, angiotensin II receptor blocker or angiotensin-converting enzyme inhibitor, beta-blocker, calcium channel blocker, furosemide or hydrochlorothiazide, insulin, and oral antihyperglycemic agent. Abbreviations: cytokine-induced neutrophil chemoattractant 1 (CINC-1), cluster of differentiation 95 (CD95), and confidence interval (CI).

## Data Availability

The original contributions presented in the study are included in the article. Further inquiries can be directed to the corresponding author.
